# Contingency-based emotional resilience: effort-based reward training and flexible coping lead to adaptive responses to uncertainty in male rats

**DOI:** 10.3389/fnbeh.2014.00124

**Published:** 2014-04-28

**Authors:** Kelly G. Lambert, Molly M. Hyer, Amanda A. Rzucidlo, Timothy Bergeron, Timothy Landis, Massimo Bardi

**Affiliations:** Department of Psychology, Randolph-Macon CollegeAshland, VA, USA

**Keywords:** resilience, uncertainty, prediction errors, DHEA, contingency training, neuroplasticity markers, coping profiles

## Abstract

Emotional resilience enhances an animal's ability to maintain physiological allostasis and adaptive responses in the midst of challenges ranging from cognitive uncertainty to chronic stress. In the current study, neurobiological factors related to strategic responses to uncertainty produced by prediction errors were investigated by initially profiling male rats as passive, active or flexible copers (*n* = 12 each group) and assigning to either a contingency-trained or non-contingency trained group. Animals were subsequently trained in a spatial learning task so that problem solving strategies in the final probe task, as well-various biomarkers of brain activation and plasticity in brain areas associated with cognition and emotional regulation, could be assessed. Additionally, fecal samples were collected to further determine markers of stress responsivity and emotional resilience. Results indicated that contingency-trained rats exhibited more adaptive responses in the probe trial (e.g., fewer interrupted grooming sequences and more targeted search strategies) than the noncontingent-trained rats; additionally, increased DHEA/CORT ratios were observed in the contingent-trained animals. Diminished activation of the habenula (i.e., fos-immunoreactivity) was correlated with resilience factors such as increased levels of DHEA metabolites during cognitive training. Of the three coping profiles, flexible copers exhibited enhanced neuroplasticity (i.e., increased dentate gyrus doublecortin-immunoreactivity) compared to the more consistently responding active and passive copers. Thus, in the current study, contingency training via effort-based reward (EBR) training, enhanced by a flexible coping style, provided neurobiological resilience and adaptive responses to prediction errors in the final probe trial. These findings have implications for psychiatric illnesses that are influenced by altered stress responses and decision-making abilities (e.g., depression).

## Introduction

Emotional resilience, an organism's ability to respond to stressful situations in an adaptive manner, provides a neurobiological buffer against the negative effects of stress and leads to decreased susceptibility to psychiatric illnesses (Southwick and Charney, 2012; Wu et al., 2013). The classic learned helplessness research assessing dogs' responses in stressful contexts emphasized the relevance of perceived controllability in emotional resilience. In these early studies, inactivity was viewed as a result of perceived uncontrollability and, subsequently, became a popular model of depression (Overmier and Seligman, 1967; Seligman and Maier, 1967; Abramson et al., 1978). In addition to controllability, cognitive factors such as cognitive reappraisal and cognitive flexibility have emerged more recently as important factors in emotional resilience (Feder et al., 2009; Koolhaas et al., 2011; Colby and Shifren, 2013; McRae et al., 2012).

Extending from the learned helplessness research and focusing on decision-making in the presence of uncertainty, the role of prediction errors has been explored. When an anticipated outcome is not realized (i.e., there is a discrepancy between an expected and observed outcome), also known as a *prediction error* in these contexts (Bubic et al., 2010; Robinson et al., 2013; Steinberg et al., 2013), it is adaptive for the brain to update relevant contingency probabilities. The uncertainty that accompanies prediction errors serves as a neural prompt for relevant probability upgrades, even when the prediction error occurs in the midst of well-learned response-outcome contingencies (Rushworth and Behrens, 2008). If a response is ultimately perceived as being influential in the production of an anticipated outcome, then an animal has a heightened sense of controllability (Moore et al., 2009). Accordingly, stronger perceived contingencies lead to a heightened capacity for goal-directed behavior (Liljeholm et al., 2011). For example, contingency management is a critical component of Behavioral Activation psychotherapy, an empirically supported therapy for depression (Dimidjian et al., 2011).

Several brain areas have been implicated in both contingency building and contingency restructuring in the presence of prediction errors. For example, the anterior cingulate cortex (Ragozzino and Rozman, 2007; Rushworth and Behrens, 2008) and the medial prefrontal cortex (Matsumoto and Tanaka, 2004; Behrens et al., 2007; Alexander and Brown, 2011; Egner, 2011) have been associated with the detection of the volatility, or uncertainty, of the parameters associated with a specific environmental context. Further, the insular cortex has been implicated in the neural processing of negative outcomes that promote negative emotional responses so that the aversive response can be avoided in the future (Endepols et al., 2010) as well as the facilitation of adaptive decisions in various challenges (Rebola et al., 2012). Finally, the retrosplenial cortex plays a role in the determination of behavioral shifts for the successful completion of emotional tasks with cognitive demands (Bush et al., 2000; Damasio et al., 2000; Vann et al., 2003, 2009; Vann and Aggleton, 2004; Pothuizen et al., 2008; Bardi et al., 2013) with lesions of this area interfering with the processing of fear-related stimuli such as electric shock (Gabriel and Talk, 2001).

It has also been suggested that the habenula, a structure characterized as an important link connecting the midbrain and forebrain in the regulation of emotional behaviors, influences decision making in uncertain contexts. Lesions of the lateral habenula in animal models result in hyperactive responses in emotional contexts; thus, this brain area may contribute to the suppression of behavioral responsiveness, such as freezing in the presence of prey, in stressful situations (Li et al., 2011). Accordingly, the lateral habenula may contribute to the diminished responsiveness observed in the previously discussed learned helplessness model of depression. In humans, the lateral habenula appears to be hyperactive in depressed patients, as well as in healthy individuals experiencing unwanted negative feedback following a failed performance (Li et al., 2011; Savitz et al., 2011; Henn, 2012).

In recent studies in our laboratory, an alternative to the learned helplessness model, that is, learned persistence, has been investigated as a model of enhanced emotional resilience in the midst of uncertainty related to response outcomes (Lambert, 2006; Bardi et al., 2012, 2013). Specifically, effort-based reward (EBR) training, in which rodents are trained to dig for food rewards in a large arena daily for several weeks, is utilized to build response-outcome contingencies. When presented with a novel, unsolvable problem-solving task, contingency-trained rats were observed to persist longer on the task than comparable noncontingency-trained animals. Further, the contingency-trained animals had higher dehydroepiandrosterone (DHEA)/corticosteroid (CORT) ratios (Bardi et al., 2012); higher levels of DHEA have been associated with adaptive behavioral responses to stress as well as neurotrophic effects (Karishma and Herbert, 2002). Considering that diminished levels of DHEA have been observed in psychiatric illness such as anxiety, depression and schizophrenia (Morgan et al., 2009), altered DHEA/CORT ratios following EBR training may have contributed to learned persistence in the aforementioned problem solving task.

When contingent-trained rats in our laboratory were exposed to a spatial learning task (the dry land maze, DLM), they also exhibited persistence in the probe trial in which the reward was removed from the previously baited well. In this task, contingency trained rats responded to the prediction error very differently than the rats receiving no contingency training. Specifically, contingency trained rats maintained the strategy of remaining in proximity to the previously baited well, both approaching it faster and spending more time interacting with it, than non-trained animals. Focusing on brain responses, even though the noncontingent-trained animals failed to exhibit an adaptive strategy (i.e., enhanced memory of previously baited well) in the probe trial, they exhibited higher fos activation in the insula, retrosplenial cortex and dentate gyrus of the hippocampus. These results suggested that higher activation in areas involved in emotional and cognitive processing accompanied less efficient responses in this task (Bardi et al., 2013).

In addition to the influence of behavioral training on aspects of decision-making in challenging situations, predisposed individual factors also play an influential role. Although the stress response was once thought to be generalized and non-specific (Selye, 1936), it has become increasingly clear that individual differences in stress responsiveness exist. For example, varying responses to stressors, or coping styles, have been identified among members of the same species (Koolhaas et al., 2007). Coping strategies are considered effective when they provide neurobiological adaptations that mitigate threat and enhance survival in threatening situations (Wechsler, 1995). When approach responses to novel objects are assessed in young rats, some animals readily approach the objects whereas some avoid the novel objects. Interestingly, uninhibited rats experience a faster recovery of corticosteroids following a stressful encounter, suggesting that they are less likely to experience allostatic load and an unhealthy stress response. Because the responses of these animals remained consistent when assessed at different developmental stages, these coping response strategies are viewed as an emotional trait that is persistent across the animal's lifetime (Cavigelli and McClintock, 2003; Cavigelli et al., 2007).

Building upon research determining passive and active coping styles in piglets (Koolhaas et al., 1999), a back-test restraint assessment was adapted for rodents to determine predispositions for both consistent and variable, or flexible, coping strategies (Lambert, 2006; Bardi et al., 2012, 2013). In one study, rats were profiled as active, passive and flexible copers and exposed to 3 weeks of chronic unpredicatable stress. Focusing on apparent neurobiological adaptations, the flexible coping rats had higher levels of Neuropeptide Y (known for its role in emotional resilience) in the basolateral amygdala and bed nucleus of the stria terminalis (Hawley et al., 2010; McGuire et al., 2011). Subsequent research in our laboratory has also confirmed the role of hippocampal NPY–immunoreactive cells in various coping profiles with flexible copers exhibiting higher levels in the CA1 subfield than their passive and active counterparts (Bardi et al., 2012).

Taking these findings into consideration, the purpose of the current study was to investigate behavioral and neurobiological responses in rodents exposed to uncertainty in a decision-making task. Of interest was the animal's response to uncertainty related to a prediction error in the cognitive task and the degree of response flexibility, a response that has been interpreted as adaptive in such situations (Donegan et al., 2013; Klanker et al., 2013; Rivalan et al., 2013). Specifically, male Long-Evans rats were profiled as active, passive or flexible copers then exposed to either 4 weeks of EBR contingency training or noncontingency training. Following contingency training, both contingent and noncontingent groups were trained in the Dry Land Maze so that, following learning acquisition, decision-making strategies could be observed in response to a prediction error (i.e., negative surprise) in the probe trial. In addition to relevant behavioral responses, the animals' brains were assessed for various forms of activation throughout several brain areas known to facilitate strategic decision-making in emotional contexts. Further, with the established role of neuroplasticity in both cognitive responses and emotional resilience (e.g., protection against depression symptoms), neuroplasticity in various hippocampal areas was also assessed in the current study (Krishnan and Nestler, 2013). It was hypothesized that predisposed flexible coping strategies, as well as contingency training, would result in more adaptive decision-making strategies (in this case, the presence of varied and flexible responses) and neurobiological responses consistent with enhanced survival (e.g., avoiding threats and excessive allostatic loads).

## Methods and materials

### Animals

**Figure 1:**
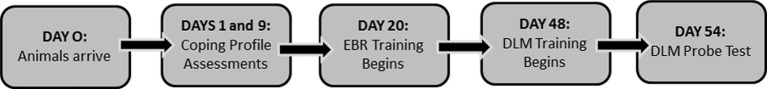
**Timeline for behavioral procedures utilized in the current study**. Animals arrived in the laboratory at approximately 23 days of age then were exposed to the sequence of events in this timeline.

Fifty male Long-Evans rats were ordered from Harlan Tekland (Madison WI USA) and arrived at 21–23 days of age. At the time of arrival they were housed five animals per cage (48 × 26 × 21 cm) with corncob bedding and food and water provided *ad libitum*. A 12 h: 12 h light dark schedule was maintained with lights on at 6:00 am. Following 17 days of habituation to the laboratory, animals were matched with a novel cage-mate with the same coping profile (active, passive or flexible; see below for coping profile assessment). At that time one of the two animals was assigned to the EBR (EBR) contingency-training group (C-T) and one to the control non-contingent training group (NC-T). See Figure 1 for timeline of behavioral procedures described below.

### Coping profile assessment

Two days following arrival to the laboratory, coping profile assessments were conducted in the colony room between the times of 1:30 and 3:30 pm with each session videotaped for subsequent confirmation of behavior. During the assessment, each animal was gently restrained on its back for 1 min so that the number of escape attempts (or wiggles) could be quantified (see Hawley et al., 2010). Seven days later the same assessment was conducted; however, the animals were tested in a different order than the first assessment to avoid any confounding sequencing effects. Once the two sessions were observed and scored, the total number of escape attempts was used as the criterion score for the determination of coping profile. Considering that the greatest number of escape attempts for each session was 11 responses, animals with fewer than 6 responses were categorized in the passive coping group and those with 6 or more responses were categorized as active copers. If the response number stayed in the same category in the second assessment 1 week later then its final placement was in that respective category. However, if the animal switched from one category to another (in either direction), it was classified as a flexible coper. The most representative animals were selected from the 50 animals so that each group consisted of 5–6 animals with the lowest and highest escape attempts (passive copers and active copers, respectively); accordingly, the flexible copers with the largest differences in escape attempts were assigned to the flexible coping groups. In total, 34 animals placed in six groups (two training groups × 3 coping groups) were used in the current study. Animals were maintained in accordance with the Randolph-Macon College Institutional Animal Care and Use Committee.

### Effort based reward (EBR) training

Half of each coping group was assigned to the contingent-trained (C-T) group and half to the noncontingent-trained (NC-T) group. Further, half of the animals in each group commenced training 1 week later than the other animals to evenly space out training, testing, and histological processing for the investigators. Twenty days after arrival to the lab, the first group of animals commenced training. Food was removed from the cages at 9:00 am on the first day of training and each rat received a piece of sweet cereal (i.e., froot loop® cereal) to habituate to the taste of the food reward used in the task (see Bardi et al., 2012, 2013). Approximately 4 h later, training began by placing the rats individually into an open arena (122 × 91 × 51 cm) built of wood and covered with gray linoleum. Corn-cob bedding was placed on the floor of the arena at a depth of approximately 7 cm. For the C-T group, the bedding was arranged into four mounds that were approximately 10 cm tall with the locations of the mounds varied each day. In the early trials, a half piece of cereal was placed on the top of the mound so that it could be easily detected by the rats. The C-T animals were subsequently placed in a consistent corner of the arena and were given 6 min to explore the arena and mounds with food rewards; however, if all the froot loops were retrieved prior to the 6 min period, the animal was removed from the arena so it wouldn't experience the mounds with no rewards and compromise the contingency associations between digging and rewards. Following training, each animal was weighed and placed in its home cage. Between sessions, the bedding was redistributed to mask the previous animals' scent and paths for the subsequent N-CT animal. Each N-CT animal was time-yoked to the C-T animal and placed in the arena with mounds but the cereal rewards were placed collectively in the open so that the N-CT could access the rewards with minimal effort. After the first week, the N-CT animals were also yoked to the C-T animals on the number of rewards consumed so that, after week one, the C-T and NC-T animals were both time- and reward-yoked. Also beginning during the second week of training, the cereal pieces were slightly hidden in the top of each mound, then at the beginning of the third week the rewards here hidden 2.5 cm in the mounds and, by the fourth week, they were positioned near the bottom of the mounds. During training, since the food was removed for approximately 4 h each day (with food available *ad libitum* for the rest of the day), the animals were weighed every third day and monitored for appropriate growth to assure that they were maintaining healthy weights with no animal exceeding more than a 10% weight loss. This training continued for 4 weeks (with no training on weekend days).

### Dry land maze (DLM) training and problem solving assessment

On the day following the completion of EBR contingency training, animals commenced habituation training for the DLM task (see Franssen et al., 2011; Bardi et al., 2012). During this training the same food restriction protocol as the EBR training was used and time of testing was shifted 2 h later. A circular apparatus measuring 124.5 cm in diameter and 40.5 cm in height was used for training and testing. Eight plastic wells (2 cm in diameter and 1 cm in height) were secured on the bottom of the arena positioned equidistantly along the periphery. During habituation day 1, all 8 wells were baited with one-quarter of a cereal piece. Animals were once again given 6 min to locate and consume the food rewards. On days 2 and 3 of habituation training, four wells (every other one) and two wells (two of the four) were baited, respectively, for the 6 min trials. On the following day, animals were exposed to the Acquisition Trial which consisted of only one of the two previously baited wells being baited for the testing and probe trial assessment. For the three subsequent test days, the same single well was baited and each animal was exposed to three 3-min trials with an inter-trial interval of 1 min. Animals were placed in varying start positions throughout the test trials; however, the start positions were consistent for all animals. On the day following the second day of testing, animals were exposed to the probe trial to assess reactions to prediction errors by being placed in the DLM arena for 5 min with no reward in the previously baited well. Each animal's behavior was videotaped for subsequent behavioral analysis of the following behaviors: latency to approach the previously baited well; time spent in proximity to the baited well; rearing; investigation of other wells and freezing responses (see Table 1 for ethogram of behaviors scored in the probe trial). Together with these traditional behavioral measures, we also recorded behavioral sequences and transitions for both movements and grooming. Distractions or interruptions of sequenced behavioral responses have been associated with heightened anxiety (Bardi et al., 2011). The common and widespread use of averaged measures taken at specific time intervals is useful for collecting snap-shots summaries of behavior, but they often fail to disentangle more subtle variations in behavior (Bardi et al., 2011). Therefore, we calculated the number of interrupted grooming sequences (either an incomplete chain or a chain diverging from the typical cephalocaudal structure), the number of inversions (i.e., changes in directions while in movement such as walking from clockwise to anticlockwise), the number of stereotypical movements, and the number of behavioral transitions (e.g., from running to exploring).

**Table 1:** **Ethogram of the behavioral measures**.

**Behavioral measure**	**Definition**
Freezing	Suddenly becoming rigid or motionless. Fear reaction
Exploration	Information gathering necessary for the formation of spatial representation (touching or sniffing the environment)
Scratching	Rapid movements of hand or foot to rake or pick the skin of the body
Grooming	Complex strings of movements to clean and maintain the fur and skin of the body (wiping, licking, and strokes). Sequential chain
Grooming interrupted	Non-chain sequences of grooming (incomplete sequences or random sequences)
Rearing	Standing up on the hind legs sniffing
Proximity	Closer to one body length to the previously baited well
Jumping	Sudden movements spring of the ground
Inversion	Any change in the direction of movement
Stereotype	Any form of rapidly repeated movement
Transition	Any change in behavior (i.e., from freezing to exploring)

### Physiological responses

To assess the physiological responses to the behavioral tests, CORT and DHEA metabolized in excreta were assessed using fresh fecal samples. Samples were collected at approximately 9:00 am with the baseline sample collected during the second week of EBR training (following a weekend nontraining day with no food restriction) and, for the test sample, 12 h following the initial acquisition trial of the DLM, representing a challenging phase of the DLM training (see Bardi et al., 2011 for parametric validation of times used in fecal sample collection). Thus, the test samples were collected following a rather mild cognitive challenge phase of the DLM task and were not perceived as fully engaged stress responses. Animals were briefly isolated (no more than 10 min for each animal was necessary to collect samples) and samples (0.1 g each) were collected from each animal and frozen unmixed in sealed containers at −70°C until assaying. A total of 48 samples were collected and saved for CORT and DHEA extraction and assay procedures.

Prior to extraction, previously collected fecal samples were thawed at room temperature and placed in a glass tube with 1 ml of 100% methanol. The contents of the tube were then mixed via vortex (Vortex Genie 2, Scientific Industries, Inc.) for approximately 30 s. Next, the tube was centrifuged for 10 min at 2500 rpm. Using a transfer pipette, the sample was transferred to a 13 × 100 mm glass test tube. The final step of extraction procedures was to dilute the sample in MeOH (concentration 1:20) in an EIA buffer. Assay procedures were carried out using materials and protocols provided by an Enzyme ImmunoAssay (EIA) kit (Assay Designs, Anne Arbor, Michigan). Correlate-EIA sample readings were completed using an automated micro-plate reader (BioTek, Winooski, VT, model # EL × 800) and the KC Junior software (BioTek, Winooski, VT, version 1.3, Part 5270501). Readings were assessed at a wavelength of 405λ. The cross-reactivity of the cortisol kit, as reported by the manufacturer, was 100% with cortisol and prednisolone (122%), 27.68% with corticosterone, 4% with 11-deoxycortisol, and negligible for other steroids (under 1%). The cross-reactivity of the DHEA kit was 100% with DHEA, 30% with DHEA sulfate, and considered negligible for other steroids (under 1% of androstenedione, androsterone, and so forth). Sensitivity of the kit was 56.80 pg/mL for cortisol and 2.90 pg/mL for DHEA.

To demonstrate parallelism and accuracy of the CORT and DHEA assays, each of five randomly selected fecal samples were serially diluted (1:2–1:16). Percentage-binding data from the standard curve were plotted against logarithmic transformations of their dosages and the resulting regression equation was compared to those of the dilution sequences. To assess dose response, another five samples were randomly selected and unlabeled CORT or DHEA was added to 10 L sample aliquots in increasing doses: 0, 2.5, 10, 40, and 160 pg/tube.

The dose response study generated a curve with a slope of 1.03 (*r*^2^ = 0.975) for CORT and a curve with slope of 1.01 (*r*^2^ = 0.997) for DHEA. Mean recoveries were 95.21% for CORT and 97.19% for DHEA over a range of 2.5 to 160 pg. The slopes generated from the serially diluted samples in the parallelism study were not different from the standard curve slope (CORT: *p* = 0.866, DHEA: *p* = 0.815). Intra- and interassay coefficients of variations were 4.43 and 11.15% for CORT and 5.33 and 9.87% for DHEA.

### Histological preparation

Ninety minutes following the 5-min probe trial, animals were anesthetized and perfused to detect fos-immunoreactivity as well as other relevant neural markers. Specifically, individual animals were exposed to 1 mL of Halothane liquid (Sigma-Aldrich; St. Louis MO) until respiratory rate slowed and were subsequently given an intraperitoneal injection of 0.2 mL sodium pentabarbitol at an overdose of 50 mg/Kg and transcardially perfused at 40 mL/min using a MasterFlex L/S perfusion pump initially with 100 mL phosphate-buffered saline solution, then with 200 mL 4% paraformaldehyde. Following extraction, brains were post-fixed in 4% paraformaldehyde overnight at 4°C, then transferred to a 10% sucrose solution for 24 h at 4°C followed by a 20% sucrose solution at 4°C until the time of sectioning. Brains were subsequently sectioned at −25°C using a HM525 Microm cryostat at the appropriate Bregma position for each targeted brain (using Paxinos and Watson, 2007). For all sections, every 7th section was used to avoid double-counting cells in serial sections and to provide enough tissue for all the histological protocols.

**Figure 2:**
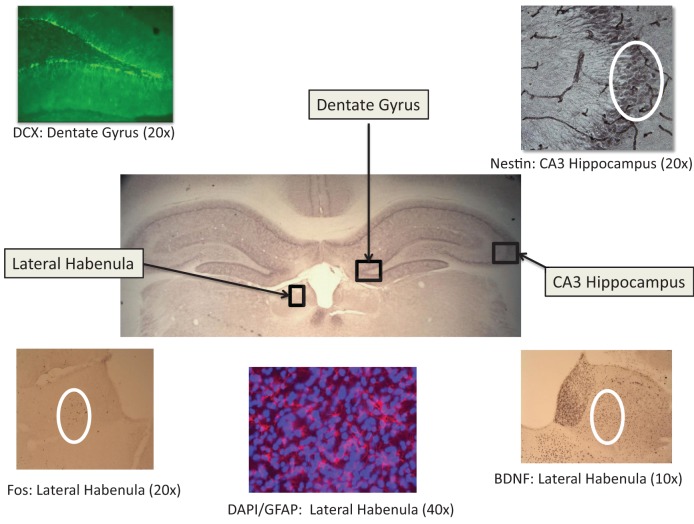
**Representative photomicrographs of relevant immunoreactive tissue in the lateral habenula and hippocampus (dentate gyrus and CA3 area**. See Results for statistical findings for each area.

Six free-floating sections (40 μm thickness) were collected through the following brain areas: Lateral habenlua; hippocampus (dentate gyrus, CA1, CA2, and CA3); and cortex [insular, restrosplenial, and piriform areas (the piriform cortex was assessed due to its projections to both the limbic and cortical structures; Johnson et al., 2000)]. See Figure 2 for brain areas and representative photomicrographs of habenula and hippocampal neural markers. For fos-immunreactivity assessment in the habenula, insula and piriform cortical areas, following a 10 min wash in 0.1% hydrogen peroxide to quench endogenous peroxidase activity, sections were blocked for 30 min in 10% normal goat serum (Vector, Burlingame, CA) in PBST (0.3% Triton-X, Spectrum Chemical: Cardena, CA). Sections were subsequently incubated in c-fos primary antibody (1:10,000; Immunostar, Inc., Hudson, WI, USA) overnight at 4°C followed by 1 h exposure to goat anti-rabbit secondary antibody (Vector) at 1:1500, and further processed with a standard Vectastain ABC kit (modified with Bovine Triton-X PBS; Vector). A similar protocol was used for BDNF (Abcam, Inc., Cambridge, MA USA; primary 1:1000 and secondary 1:250) and nestin (primary antibody dilution of 1:400 and secondary dilution of 1:200; Developmental Studies Hybridoma Bank, University of Iowa, Iowa City, IA USA) to assess neurotrophic activity and neuronal restructuring (Hendrickson et al., 2011), respectively. All sections were visualized with DAB peroxidase substrate and then cleared through a series of 70, 95, and 100% EtOh and Citrisolv (Fisher Scientific, Fair Lawn, NJ, USA) washes and coverslipped using Permount (Fisher Scientific).

For fluorescent immunocytochemistry, sections were incubated in 0.3% trisodium citrate solution for 10 min in a 90°C water bath. Following three 5-min PBS-Tween washes, sections were exposed to 6% normal donkey serum in PBS-Tween for 30 min (Spectrum Chemical, New Brunswick, NJ, USA), and then incubated overnight at 4°C in a 1:1000 dilution of rabbit anti-DCX (Abcam, Inc., Cambridge, MA, USA). Following three PBS-Tween 20 min washes, sections were exposed to the secondary anti-rabbit antibody at a dilution of 1:200 (Abcam). GFAP sections were treated in a similar manner but exposed to primary anti-rabbit antibody at 1:1000 dilution and secondary rhodamine red anti-rabbit antibody at 1:200 (Abcam, Inc.). GFAP slides were counterstained with DAPI (0.01%; Abcam) for 1 h in the dark.

### Neural quantification

Prior to being quantified, all slides were recoded so that experimenters would be blind to experimental conditions. A Zeiss Axioskop light microscope (Carl Zeiss, Oberkochen, Germany) and Neurolucida software (Microbrightfield, Inc., Williston, VT, USA) were used to quantify fos-immunoreactive cells in the lateral habenula (at 10× magnification with a 300 × 300 μm grid), the piriform cortex (at 10× magnification with a 500 × 300 μm grid), and in the insula (500 × 500 μm at 10×). In these areas, cells were marked with a computer-generated colored symbol and quantified for each section.

For the remaining markers, a BA400 light microscope (Motic, Richmond, BC, Canada) was used for the standard neuroquantification of BDNF (using a 125 μm radius circle at 20× magnification) and CA1, CA2, and CA3 nestin (using a 135 × 135 μm area at 40× magnification) as well as for fluorescent neuroquantification for doublecortin in the dentate gyrus (270 × 270 μm at 10× magnification) and GFAP and DAPI in the retrosplenial cortex (135 × 135 μm at 40× magnification). In all cases, quantification was accomplished using light-thresholding software (Bioquant Life Sciences, Nashville, TN, USA) so that the proportion of positively stained tissue to nonstained tissue could be determined for each visual field. For the determination of nestin-immunoreactive cells, the nestin-immunoreactive blood vessels were subtracted from the visual field so that only pyramidal neuronal cells were quantified.

### Statistical analysis

For all dependent variables, a 2 × 3 General Linear Model (GLM) analysis was used to determine the effects of EBR training (two levels: contingent training present or not) and coping profile (3 levels: active, passive and flexible) respectively. For each analysis, the α-value was set at 0.05. Following the GLM analyses, appropriate Tukey *post-hoc* tests and Pearson's correlations were conducted to further elucidate relationships among dependent variables.

A multi-dimensional scaling (MDS) analysis was conducted in order to provide a model of independent associations among the variables. We preferred MDS to other multivariate techniques, such as MANOVA or factor analysis, since MDS does not require the data to be multi-normally distributed, a condition rarely satisfied with behavioral measures. MDS is a data reduction technique used to uncover a “hidden structure” to a set of data (Kruskal and Wish, 1978). MDS refers to graphical models that provide a spatial representation of the similarity structure of variables. Using correlations, the relationships (i.e., proximities) among variables can be displayed graphically. Because the variables are represented by a set of points in a two or higher dimensional space (a map), the closer two or more variables are on the map, the more highly correlated they are, while the farther apart they are, the less correlated they are. In order to map all of the variables into a desired space (two dimensional or greater), a certain lack of fit is accepted. This lack of fit is referred to as the s-stress. The values of s-stress range from 0 (perfect fit) to 1 (worst possible fit). Thus, the aim of MDS is to find a map of the variables that minimizes the s-stress for a given number of dimensions. The number of dimensions can be linked to the number of latent underlying factors in the dataset. Thus, when choosing the number of dimensions to represent the data, one must consider (1) the number of variables in the model, (2) the lack of fit (s-stress value), given the number of dimensions, (3) an index of fit of the model (*r*^2^-value), and (4) interpretability of the dimensions (Manly, 1994). The first point addresses the fact that for each dimension of the data, there should be approximately 4 variables entered into the model. Thus, for a 2-dimensional map, approximately 8 variables should be used. The second point addresses how well the MDS map actually “fits” the data. Stress values below 0.15 are typically deemed acceptable. The third point addresses the variance accounted for within the model. As is the case with any regression analysis, the amount of variance being accounted for is an important consideration. Typically, *r*^2^-values of 0.8 or higher are desirable. Finally, one must select a solution based on interpretability of the dimensions. Parsimony is crucial to interpreting the “map” of any given dataset. In sum, MDS is a technique that provides additional information about the “structure” of a dataset, which is not possible with standard parametric statistical techniques.

## Results

### Behavioral results

**Figure 3:**
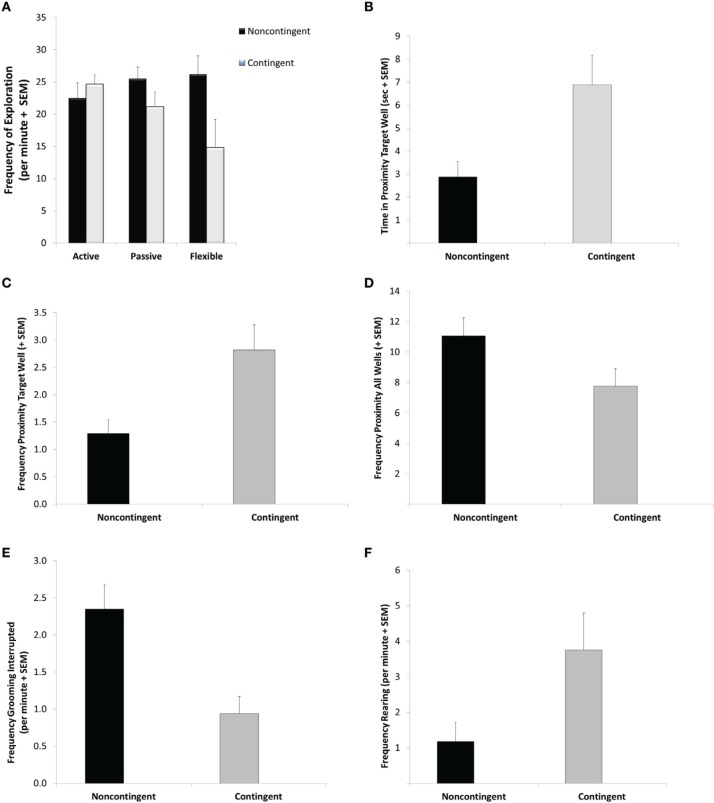
**Probe trial behavior**. During the 5-min probe trial flexible contingent animals engaged in less exploratory behavior **(A)** further contingent animals spent more time in proximity to the previously baited well **(B)** visited the previously baited well more frequently **(C)** visited the non-baited wells less frequently **(D)** exhibited fewer interrupted grooming sequences **(E)** and more rearing responses **(F)** than their noncontingent counterparts. See Results for specific statistical outcomes.

For the variable of frequency of exploration bouts during the DLM probe test, the GLM revealed a significant interaction between EBR training and coping profile [*F*_(2, 28)_ = 3.38; *p* = 0.048]. Specifically, as seen if Figure 3A, flexible C-T rats exhibited fewer exploration bouts than flexible NC-T rats (Tukey *post-hoc* test: *p* = 0.03). Additionally, a significant main effect for EBR indicated a lower overall frequency of exploratory bouts for the contingent trained animals [*F*_(1, 28)_ = 4.64; *p* = 0.04]. No differences were observed in the overall duration of exploration [corrected model: *F*_(5, 28)_ = 0.24, ns].

Focusing on the duration of time spent in proximity to the previously baited well in the DLM Probe test, a main effect for EBR training was observed [*F*_(1, 28)_ = 7.69; *p* = 0.01]. Specifically, C-T animals spent 140% more time than NC-T rats in proximity to the targeted well (see Figure 3B). C-T animals also visited the previously baited well significantly more often than the NC-T animals [*F*_(1, 28)_ = 7.297 *p* = 0.012; see Figure 3C]. Considering all of the eight wells in the DLM arena, the NC-T rats visited a higher number of wells in the arena than the C-T animals [*F*_(1, 28)_ = 4.29; *p* = 0.048; NC-T animals visited 11 wells compared to 7.6 for C-T animals; see Figure 3D].

Although no differences were found in the total duration or frequency of self-grooming a significant effect of EBR training was found in the frequency of interruptions in the grooming sequence [*F*_(1, 28)_ = 11.18; *p* = 0.002]. As seen in Figure 3E, the N-CT animals were more than twice as likely to exhibit an interrupted grooming sequence. EBR training was also found to have an effect on rearing responses in strategic areas of the DLM arena, namely in either the center or in proximity to the wells [*F*_(1, 28)_ = 4.65; *p* = 0.04; see Figure 3F]. In this case, the C-T rats exhibited approximately three times more rearing responses than their NC-T counterparts. Neither EBR training nor coping profile affected the remaining variables that were scored, including freezing, jumping, scratches, and stretch-attend responses (all *p*-values > 0.18).

### Endocrine results

**Figure 4:**
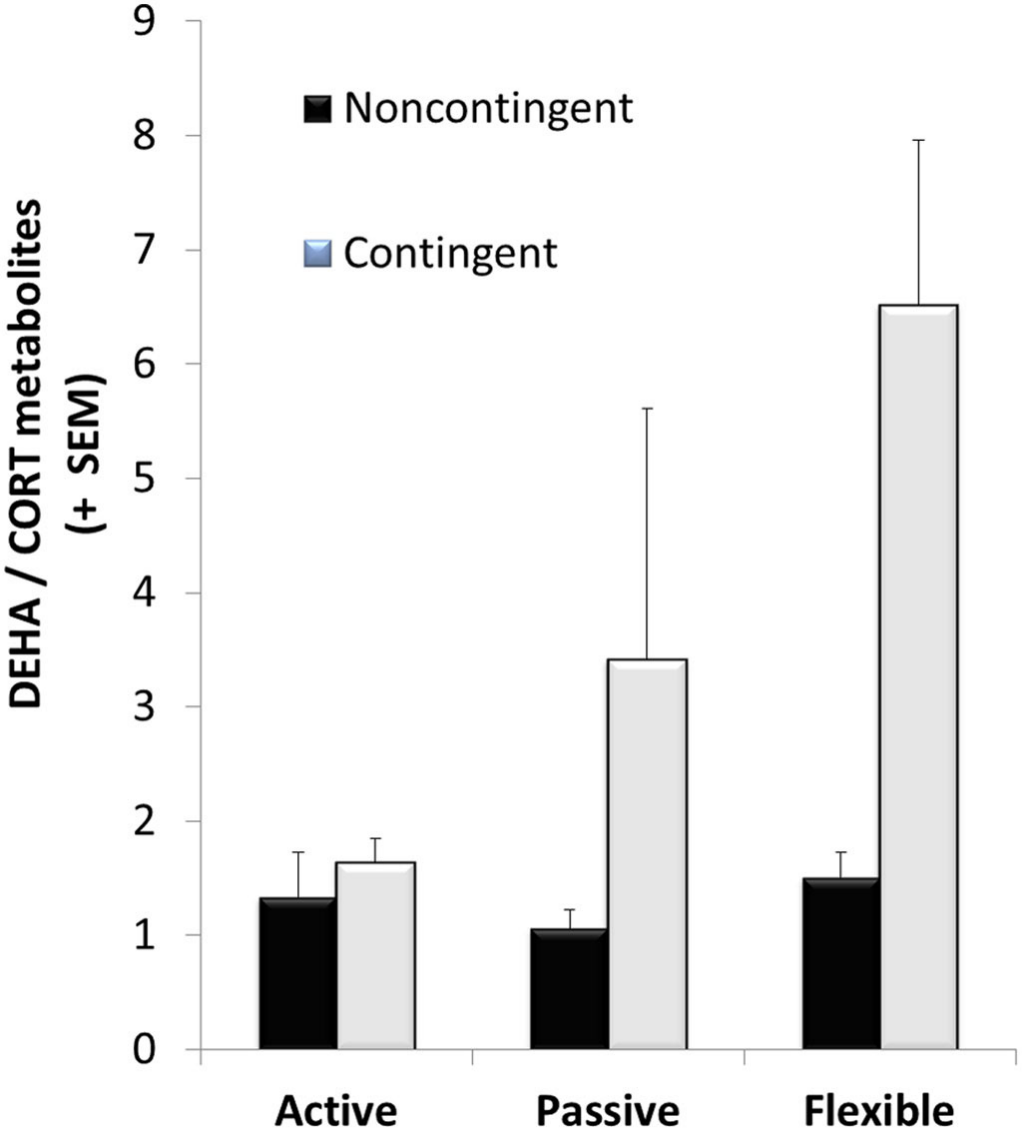
**DHEA/CORT metabolite ratios were differentially affected after the acquisition trial in the dry land maze; although there was high variability in the varying coping groups, only a significant main effect for contingency training was observed (with mean values of 3.69 ± 1.11 and 1.29 ± 0.16 for the contingent and noncontingent groups, respectively)**. Although no significant interaction effect was observed, planned comparisons between contingent and noncontingent groups for each coping style revealed a significant training effect in the flexible coping group.

CORT metabolites decreased significantly between the baseline levels collected at the beginning phases of EBR training and after EBR training following the acquisition trial of DLM testing, representing a phase of more challenging cognitive training [*F*_(1, 28)_ = 7.08, *p* = 0.013]. In contrast, DHEA metabolites significantly increased between the baseline levels to the DLM trial [*F*_(2, 18)_ = 6.60, *p* = 0.023]. After the DLM acquisition trial, DHEA/CORT ratios were calculated and C-T animals had a significantly higher ratio than the NC-T rats [*F*_(1, 28)_ = 5.57; *p* = 0.026]. Although no significant interaction was observed, planned comparisons revealed a significant effect of training in the flexible training group with C-T animals having a higher ratio than NC-T animals [*t*_(8)_ = 2.56; *p* = 0.03]. See Figure 4 for the DHEA/CORT metabolite levels in the test sample following the acquisition trial in the DLM.

### Neural results

**Figure 5:**
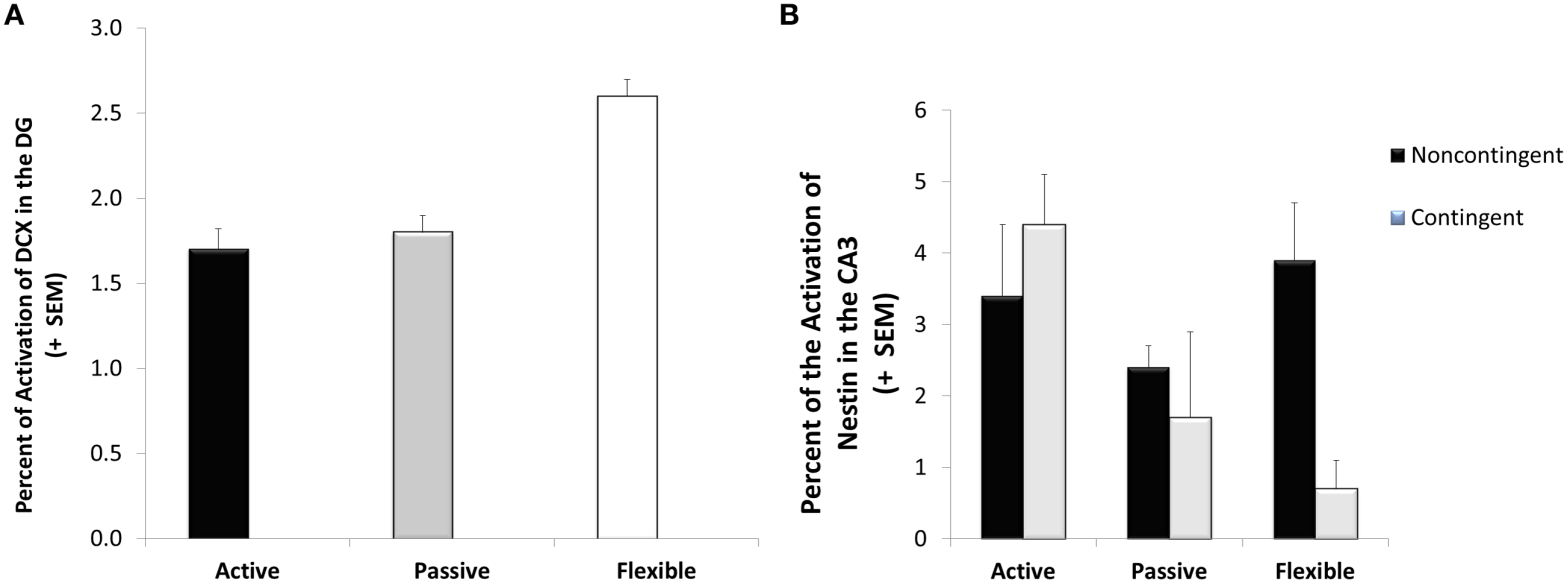
**Neuroplasticity markers**. Doublecortin-immunoreactive tissue was higher in the flexible coping animals than their active and passive counterparts **(A)** whereas a significant interaction was observed in the Nestin-immunoreactive tissue **(B)**; specifically, lower levels were observed in the flexible contingent trained animals than in the flexible noncontingent animals, an effect not observed in the active and passive coping groups.

A significant coping effect was observed in the percentage of doublecortin-immunoreactive tissue in the dentate gyrus visual field [*F*_(2, 28)_ = 7.55; *p* = 0.002]. As seen in Figure 5A, the flexible coping animals had about 66% more DCX-immunoreactive tissue than the passive and active groups. Focusing on another marker of neuroplasticity, a significant interaction was observed in CA3 nestin [*F*_(2, 28)_ = 3.82; *p* = 0.034]. In this case, whereas contingency training didn't impact the active and passive coping groups, it reduced nestin-immunoreactivity in the flexible animals (see Figure 5B). Although not reaching statistical significance [*F*_(2, 28)_ = 2.49; *p* = 0.106], flexible animals had higher neuron/glia ratios in the lateral habenula (13% higher than active copers and 21% higher than passive copers).

Whereas no significant effects of EBR training or coping profiles were observed in the fos-immunoreactive data taken from the habenula, insula and piriform cortex, a few interesting significant correlations between fos-immunoreactivity in these brain areas and the neuroplasticity measures (i.e., nestin in CA2, BDNF in habenula and DCX in the dentate gyrus), as well as with the DHEA/CORT ratio data, were observed (see Table 2). The endocrine data in Tables 2 and 3 represent levels following the cognitively challenging acquisition trial in the DLM training. The DHEA/CORT ratio was positively correlated with DCX-immunoreactive cells in the dentate gyrus (*p* = 0.04); further, a nonsignificant trend was observed suggesting an inverse correlation between DHEA/CORT ratios and fos-immunoreactive cells in the habenula (*p* = 0.09). Focusing on the CORT levels, a significant positive correlation was observed with the nestin CA2 fos-immunoreactive cells (*p* = 0.05); further, a nonsignificant inverse relationship was observed between immunoreactive-BDNF tissue in the lateral habenula and CORT (*p* = 0.06). Finally, a significant negative correlation was observed between DHEA levels and fos-immunoreactive cells in the lateral habenula (*p* = 0.03).

**Table 2:** **Correlations among neural activation and hormonal metabolites levels after the dry land maze (DLM) acquisition trial**.

**Hormone levels after DLM acquisition trial**	**CORT**	**DHEA**	**DHEA/CORT**
DCX-ir (DG)	−0.193	0.166	0.358^*^
	ns	ns	(*p* = 0.037)
Nestin (CA2)	0.341^*^	0.052	−0.203
	(*p* = 0.048)	ns	ns
Fos-ir (habenula)	−0.206	−0.373^*^	−0.292^∧^
	ns	(*p* = 0.030)	(*p* = 0.094)
BDNF (habenula)	−0.326^∧^	−0.122	−0.011
	(*p* = 0.060)	ns	ns

* p-value < 0.05;

∧ *p-value < 0.10*.

**Table 3:** **Corticosterone, DHEA, and DHEA/Corticosterone ratios by coping and training**.

**Hormone levels after DLM acquisition trial**	**CORT (1.1 g/0.1 g feces)**	**DHEA (pg/0.1 g feces)**	**DHEA/CORT**
Flexible	834	1835	4.008
Active	886	1192	1.481
Passive	872	1305	2.235
Contingent	606	1522	3.695
Non-contingent	1125	1320	1.286

### Integrative multidimensional scaling map

**Figure 6:**
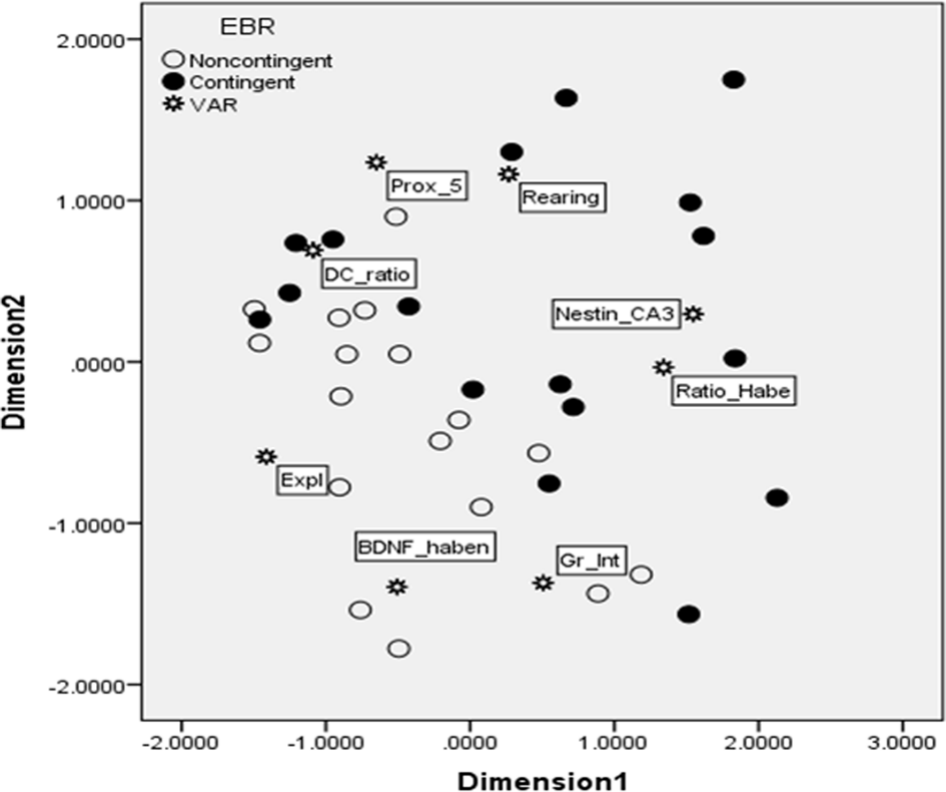
**Multidimensional scaling map exhibiting proximity of key variables for the contingent and noncontingent animals during the DLM probe trial**. As indicated in Results, the stress value for MDS was 0.101, and *R*^2^ = 0.956, thus indicating that the map was accurate and able to explain most of the variability. Two major clusters were clearly identified by the MDS, separated in the figure by a line. The lower quadrants represent noncontingent animals that displayed higher BDNF in the habenula (BDNF_haben), together with higher level of grooming interrupted (Gr_Int), and explorative behavior (Expl). The second group, in the upper quadrants, was characterized by contingent animals displaying higher neuron to glial cells ratio in the habenula (Ratio_Habe), lower nestin activation in the CA3 contingent flexible animals (Nestin_CA3), a higher DHEA/CORT ratio (DC_ratio), more time spent in proximity of the previously baited well #5 (Prox_5), and rearing (Rearing).

To take into account the multivariate association among measures assessing different systems (neural, endocrine, and behavioral measures) a multidimentional scaling (MDS) analysis was used. The MDS technique generated Figure 6. The Kruskal's stress index determined a stress value equal to 0.101, indicating a good fit between the dimensions and the mapped distances. The *R*^2^-value designated that 95.6% of the variance in the data was explained by the model. Based on the two dimensions, the responses to the EBR conditions were divided into two major clusters, indicated in the figure by a line. The lower quadrants represent noncontingent animals that displayed higher BDNF expression in the habenula (BDNF_haben), together with higher level of grooming interrupted (Gr_Int), and explorative behavior (Expl). The second group, in the upper quadrants, was characterized by contingent animals displaying higher neuron to glial cells ratio in the habenula (Ratio_Habe), lower nestin activation in the CA3 (Nestin_CA3), a higher DHEA/CORT ratio (DC_ratio), more time spent in proximity of the previously baited well (Prox_5), and rearing (Rearing).

## Discussion

In the current study, both predisposed coping strategies and contingency training were utilized to determine their impact on the animals' neurobiological and behavioral responses to uncertainty related to prediction errors. The results indicated that EBR contingency training modified targeted neurobiological factors associated with enhanced emotional resilience. Further, the identification of predisposed coping strategies highlighted relevant neural responses and interactions associated with behavioral training. Corroborating previous findings in our lab, the current findings emphasize the potential value of strategic cognitive-behavioral training programs for shaping adaptive neural responses in the midst of uncertainty (Bardi et al., 2012, 2013). Among the neural responses investigated, the roles of differential expression of neuroplasticity in the hippocampus, as well as complex involvement of the lateral habenula, emerged as potentially influential responses to uncertain conditions in the current data set.

Focusing on the animals' behavior in response to the prediction error in the probe trial, the effect of EBR training (as opposed to coping strategies) had the most significant impact on the animals' responses. The C-T animals spent more time in proximity to the previously baited well and, additionally, exhibited more rearing responses in the arena. Alternatively, the NC-T animals visited more wells in the arena and exhibited an increased number of interrupted grooming bouts. The only behavioral variable impacted by coping style was exploration in the arena. In this case, coping style interacted with contingency-training so that, although training didn't differentiate the active and passive copers, it was associated with decreased exploration in the flexible copers. Thus, the behavioral data suggest that the C-T animals utilized a more strategic search strategy—targeting the most recently baited well and exhibiting more rearing responses, providing animals with an opportunity to scan the environment for potentially relevant cues from a different vantage point. Alternatively, the NC-T animals exhibited a more general, less specific search by visiting more of the wells, regardless of their reinforcement history. The effect of training on grooming suggests that NC-trained animals had a heightened stress response relative to the C-T animals (Bardi et al., 2011). Namely, increased interruptions of grooming bouts in the NC-T animals suggest that these rats possessed less emotional regulation than the contingent-trained animals. This observation is reinforced by the endocrine data collected several days prior to the probe trial, indicating that the trained animals had higher DHEA/CORT ratios, an index of emotional resilience (Karishma and Herbert, 2002; Morgan et al., 2004; Yehuda et al., 2006; Feder et al., 2009; Bardi et al., 2012). Thus, these findings indicate that, in addition to the strategic responses exhibited in the presence of uncertain contextual contingencies, the contingency trained animals demonstrated increased emotional regulation. It is important to point out that the DLM acquisition trial, although challenging in that it was the first trial with only one food reward available in the food wells, did not appear to be any more stressful than the early stages of the EBR training. Thus, it would be interesting to explore endocrine responses in more challenging situations to understand more about the effects of EBR training on allostasis and emotional regulation in various stressful contexts. A limitation of the current study was that fecal samples, which need to be collected 12 h following the event of interest to detect the endocrine peak, could not be collected following the probe trial due to the need to process the brain tissue shortly after the trial for fos-immunoreactivity.

The pattern of neural responsiveness in the varying coping and training groups, not surprisingly, is complex. Nonetheless, certain patterns emerged in the current data set. As described earlier, the lateral habenula appears to play an important role in an animal's responses to uncertainty (Li et al., 2011); however, results of the current study suggest that its impact on uncertainty in the DLM needs further exploration. A significant inverse correlation between DHEA (during the DLM acquisition trial) and fos-immunoreactivity in the habenula following the probe test indicates a lower activation in this area in the animals exhibiting the most resilient endocrinological profile. This finding corroborates previous research characterizing the lateral habenula as playing a role in behavioral suppression in the context of perceived threats (Geisler and Trimble, 2008), with increased activation leading to diminished behavioral flexibility (Geisler and Trimble, 2008; Bianco and Wilson, 2009). Additionally, although not statistically significant, flexible animals had a higher ratio of neurons to glia than the consistent passive and active copers and this variable was relevant in the MDS analysis. If corroborated in future studies, this finding suggests that, in the flexible animals, fewer glial cells relative to neurons may be present in the laberal habenula. Increased glial cells per neuron may contribute to heightened reactivity in this area, leading to disrupted emotional regulation and top–down processing necessary for strategic responses. When the neuroanatomy of the laberal habenula is considered, its relevance in the symptoms of depression becomes apparent. Processes extending from the lateral habenula to the raphe nuclei influence the serotonergic system that is so frequently implicated in depression symptoms and treatment; further, the habenular efferents exert tonic inhibition on dopaminergic neurons in the ventral tegmental area, potentially affecting pleasure and reward experiences that characterize the symptoms of depression (Lecourtier et al., 2008; Bianco and Wilson, 2009). Functionally, the lateral habenula conveys information about prediction errors to the mesolimbic dopaminergic area of the brain, leading to a significant impact on learning responses. Suppressing lateral habenula activity with a GABA antagonist causes increases in DA release in the nucleus accumbens and striatum in a similar time course and magnitude to those observed during reward-seeking behavior (Bianco and Wilson, 2009). Considering its interactions with both the serotonergic and dopaminergic systems, as well as its input from both the limbic system and the medial prefrontal cortex, the lateral habenula can be envisioned as a neuroanatomical hub of the constellation of symptoms consistent with depression (e.g., disrupted reward processing, goal-seeking, and strategic action-outcome processing) (Geisler and Trimble, 2008; Matsumoto and Hidosaka, 2009; Li et al., 2011; Henn, 2012).

Altered patterns of neuroplasticity have also been considered to be influential in the emergence of depressive symptoms. Increased BDNF (a neurotrophic factor), as well as increased rates of neurogenesis, have been associated with the effectiveness of antidepressants (Malberg et al., 2000; Santarelli et al., 2003; De Foubert et al., 2004). In the current study, two measures of neuroplasticity, including doublecortin- and nestin-immunoreactivity were influenced more by coping profiles than training. Namely, the flexible animals had higher levels of doublecortin-immunoreactivity (an index of cellular proliferation) in the dentate gyrus and the flexible contingent-trained animals exhibited decreased levels of nestin-immunoreactivity (an index of restructuring of mature cells in this case) in the hippocampal CA3 area, but not in the CA1 and CA2 areas (Hendrickson et al., 2011). These divergent findings of neuroplastic functions in the same group of animals confirm the complexity of neuroplasticity and the importance of monitoring several indices to obtain the complete picture of the brain's neuroplastic profile. It has been suggested that BDNF may be differentially involved with specific aspects of depression symptoms (Duman and Monteggia, 2006; Martinowich et al., 2007); accordingly, the coping profiles and behavioral training used in the current study provide an opportunity for systematic investigations of several measures of neuroplasticity.

Using both statistical findings in the current study and theoretical inferences based on past findings reported in the literature, a MDS model was built to further understand the relationships among the variables differentiating C-T and NC-T animals. Specifically, the strategies exhibited by the contingent animals, characterized by spending more time in proximity to the previously baited well and engaging in more frequent rearing responses, appear to be strategic responses in that they are associated with accurate memories of expected rewards (proximity to baited well) and the acquisition of increased information (increased rearing) to potentially resolve conflicts associated with uncertainty arising from the prediction error. From a neuroanatomical view, less restructuring of the mature CA3 neurons (determined by nestin-immunoreactive cells) and fewer glial cells servicing the lateral habenula neurons (suggested by the neuron/glia ratio measures), appear to be related to the strategic responses of the contingent-trained animals and, considering the training effect on the DHEA/CORT ratio, emotional regulation in this study. Thus, these results corroborate previous research suggesting that decreased habenular activity leads to enhanced goal seeking (Bianco and Wilson, 2009); however, the lack of a significant effect on fos-activation suggests that the role of the habenula in response to uncertainty in the DLM is complex. On the other hand, the noncontingent animals were associated with increased exploration across the entire arena and more interrupted grooming sequences. Although the exploration of additional wells may be adaptive in the sense that they once contained food rewards, the increased energy expenditure and disrupted grooming appeared as less targeted and efficient than the contingent-trained animals' strategies. However, additional research is necessary to further clarify behaviors that are consistent with strategic and adaptive responses in specific contexts. Finally, the close proximity of habenula BDNF reactivity with the noncontingent animals in the MDS model provides further support of the potential role of this structure, as well as the role of neuroplasticity, in emotional regulation and performance in cognitive tasks in uncertain conditions.

In sum, the current study confirms that the constellation of symptoms that have been associated with compromised emotional resilience and increased susceptibility to depression can be investigated in tasks involving emotional challenges such as uncertainty, ambiguity and prediction errors. Although cognitive in nature, the probe trial of the Dry Land maze also provides a view of various responses in the presence of uncertainty. In this case, the apparently mild level of stress associated with the absence of the expected reward allows for a more systematic evaluation of the interactions of the limbic and cognitive processes in the evaluation of changing contingencies. As reported above, these findings suggest that, although predisposed coping strategies are important and may be associated with a neural advantage (i.e., cellular proliferation) facilitating strategic decision-making, this advantage is tempered by behavioral training and an animal's relevant contingency history. Further research is necessary, however, to identify the specific neurobiological underpinnings of emotional resilience and how an organism's contingency history and predisposed coping strategies can contribute to enhanced adaptive functioning and diminished vulnerability to stress-related psychiatric diseases such as depression.

## Author contributions

Kelly G. Lambert designed the study and supervised all aspects of data collection, as well as writing the manuscript. Massimo Bardi conducted the endocrinological assays and statistical analyses. Molly M. Hyer and Amanda A. Rzucidlo conducted the behavioral training and trials, histology and neural quantification. Timothy Bergeron and Timothy Landis also contributed to the neural quantification.

### Conflict of interest statement

The authors declare that the research was conducted in the absence of any commercial or financial relationships that could be construed as a potential conflict of interest.
